# Characteristics of TCM constitutions of adult Chinese women in Hong Kong and identification of related influencing factors: a cross-sectional survey

**DOI:** 10.1186/1479-5876-12-140

**Published:** 2014-05-21

**Authors:** Youzhi Sun, Pei Liu, Yi Zhao, Lei Jia, Yanhua He, Steve An Xue, Xiao Zheng, Zhiyu Wang, Neng Wang, Jianping Chen

**Affiliations:** 1 School of Chinese Medicine, The University of Hong Kong, Pokfulam, Hong Kong, P.R. China; 2 School of Basic Medical Sciences, Jiangxi University of Traditional Chinese Medicine, Jiangxi, P.R. China; 3 The Second People’s Hospital of Shenzhen (The first Affiliated Hospital of Shenzhen University), Shenzhen, P.R. China; 4 Research Center for Differention and Development of TCM Basic Theory, Jiangxi University of Traditional Chinese Medicine, Jiangxi, P.R., China; 5 Parinama Investment Limited, Shanghai, P.R. China; 6 Hong Kong Breast Cancer Association of Traditional Chinese Medicine, 10 Sassoon Road, Pokfulam, Hong Kong, P.R. China

**Keywords:** Traditional Chinese medicine constitution, Chinese women, Influencing factors

## Abstract

**Background:**

Traditional Chinese Medicine Constitution (TCMC) refers to an integrated, metastable and natural specialty of individual in morphosis, physiological functions and psychological conditions. It is formed on the basis of innate and acquired endowments in the human life process, which can be divided into normal constitution and unbalanced ones. The aim of this study was to investigate the distribution of TCMCs of Chinese women in Hong Kong and its acquired influencing factors.

**Methods:**

Local Chinese women between 30 to 65years old, were recruited from 18 districts of Hong Kong (n = 944), and were assessed using the *Traditional Chinese Medicine Physical Constitution Scale* for their TCMC types. Social-demographic, reproductive, lifestyle, systemic health and emotional status information were collected through structured questionnaire. The associations between different independent factors and each TCMC type, as well as the complex unbalanced TCMC types were tested individually. Significant factors related to unbalanced TCMC types were identified in final models using multiple factor analysis.

**Results:**

A total of 764 (80.9%) participants were diagnosed with unbalanced TCMCs. The most common TCMC type was Qi-deficiency constitution (53.9%), followed by Phlegm-wetness (38.9%), Yang-deficiency (38.2%), Yin-deficiency (35.5), Blood-stasis (35.4) and Qi-depressed (31%) constitution. Six hundred and eleven participants (64.7%) had at least two types of combined and unbalanced constitutions. Stepwise logistic analysis indicated that poor systemic health condition (OR, 1.76-2.89), negative emotions (OR = 1.39), overweight (OR = 1.58), high educational level (OR = 1.18) and mental work (OR = 1.44) were significantly positively correlated with certain unbalanced TCMCs. Meanwhile, aging (OR, 0.59-0.73), exercise habit (OR, 0.61-0.79) and reproductive history (OR = 0.72) showed inverse associations with unbalanced constitutions. In addition, systemic health condition and emotional status, exercise habit and age were significantly associated with the combined unbalanced TCMC types.

**Conclusion:**

The majority of middle-aged Chinese women in Hong Kong had unbalanced and complex TCMCs. Qi-deficiency, Phlegm-wetness and Yang-deficiency constitutions are the most common constitutions. Poor systemic health condition, less-than-satisfactory emotional life, overweight and mental work are associated with and may be contributors for the formation of unbalanced TCMCs, while regular physical exercise was found to be a potential protective factor for unbalanced TCMCs.

## Background

Traditional Chinese Medicine Constitution (TCMC, named *Tizhi* in Chinese) refers to an integrated, metastable and natural specialty of individuals in morphosis, physiological functions and psychological conditions, formed on the basis of innate and acquired endowments in the process of life [1,2]. The TCMC not only determines the susceptibility to certain pathogens and diseases, but also closely related to the development and the prognosis of diseases [3,4].

Generally, TCMC is divided into balanced constitution also known as Normality (*Pinghe* in Chinese) constitution and Unbalanced/Biased (*Pianpo* in Chinese) constitution which can be further classified into several subtypes such as Yang-deficiency, Yin-deficiency, Phlegm-wetness, Qi-deficiency, Wetness-heat, Blood-stasis, Qi-depressed etc. [5-7]. According to TCM philosophy, Normality constitution represents an overall healthy state and people with unbalanced constitutions are prone to certain diseases (at least theoretically).

The type of TCMC formation is mainly determined by congenital endowments; nevertheless acquired factors including individual factors (lifestyle, dietary habits, emotion status, history of diseases and treatments) and environmental factors maybe also play a role in the process of TCMC development [1,8-11]. To date few clinical investigations on the influencing factors of TCMCs were conducted and when conducted they have primarily focused on one type of unbalanced TCMC or considered limited associated factors. There is a dearth of studies involving large study population and applying rigorous research design to detect the efficacy of various acquired factors on the formation of TCMCs.

From the perspective of TCM therapeutic theory, it is of great significance to preserve ones healthy based on his/her TCMC status. Finding the potential factors related to the biased TCMCs and then trying to eliminate the effects to maintain or obtain a new balanced TCMC is the ultimate goal in keeping health or prevention of diseases. For example, for a person diagnosed with Yang-deficiency constitution a long-term prescription with the function of tonifying Yang-qi is required. However, such prescriptions for a biased constitution might be invalid theoretically due to the interference of various confounding factors related to his/her constitution. Thus it is imperative to identify and understand confounding factors and decrease their influence to a minimum in preserving health.

The aim of this study was to survey TCMC types distribution of local Chinese women between 30 and 65years old in Hong Kong; and to identify factors associated with biased constitutions. The significance of this study is to provide scientific information for local middle-aged and elderly women to prevent the imbalance of their constitutions. In addition, more importantly, to provide comprehensive and new way of thinking to improve health.

## Methods

### Participants

A cross-sectional descriptive design and convenience sampling method was used in this study. The eligibility criteria were the following: 1) Chinese women; 2) Hong Kong locals; 3) 30 to 65 years of age; 4) Living in Hong Kong during past 3 years; 5) Able to understand the questions in Cantonese; and 6) no suffering from severe diseases (confined to those diseases being hospitalized or with liver/kidney function damage during past one year).

Participants were recruited from the communities of 15 districts, 5 women’s organizations and 2 universities in Hong Kong via more than 20 workshops, from June 2012 to September 2013. Any communities’ women residents and the female members of the women’s organizations attending the workshops were potential participants of the study. In total 1131 women were screened for participation and 40 refused and/or did not complete the questionnaires due to illiteracy (response rate is 96%). Of the 1091 participants approached, 147 were excluded (Twenty-two subjects had not lived in Hong Kong during past three years, Seventy-four were over 65 years old and 26 were less than 30 years old. Twenty-five participants had severe disabling diseases). A total of 944 women were the eligible as the sample of the study.

### Procedures

Prior to commencement of the study, ethical approval was obtained from the Research Ethics Board of the University of Hong Kong (UW 12–010). In our workshops, all potential participants were approached by a Chinese medicine professional who gave them an introduction on TCMC first, then a well-trained research staff gave them a brief introduction of the study. Written informed consent to participation was obtained prior to data collection which was carried out via self-administered questionnaire as well as face-to-face consultation. Finally, the interviewer checked the questionnaire so as to complement the missing data and verify the illogical data as much as possible.

In order to ensure the quality of investigation, three important steps were taken throughout the whole process of the investigation. First of all, to ensure consistency of the survey across sites and over time, all women were investigated using the same procedures and standards by the same interviewer to ensure inter-examiner reliability. Secondly, each questionnaire was checked carefully and those questionnaires missing the important items were excluded. Thirdly, to ensure data accuracy, a duplicate entry was carried out through Epidata 3.1 software (EpiData-Association, 2006), which was utilized to establish database and then the data were imported into SPSS version 16 (SPSS Inc., Chicago, IL) for further statistic analysis.

### The content of investigation

All content of investigation was listed in a structured questionnaire consisted of three main parts. The first part is socio-demographic information including age, working status, educational level, weight, height, marital status; The second part covered most factors that may influence TCMCs formation according to TCM theory such as state of health, emotional status, reproductive history, lifestyle factors such as smoking, alcohol intake and physical exercise habit which was defined as any kinds of exercise at least one time per week during past one year; The third part is the *TCM Physical Constitution Scale*. The original version of *TCM Physical Constitution Scale* was developed by Prof. Wang [12] and proved to be with good validity and reliability in previous studies [13,14]. It has been well-adopted in mainland China [15-17] and is regarded as the standard measurement of people’s TCMC types recommended by China Association of Chinese Medicine [18]. The scale consists of 60 items scored on a 5-point scale, ranging from 1 (not at all) to 5 (very much). It has nine subscales which assess one type of TCM constitution individually. A total score of each subscale were obtained by summing relevant item scores and then convert them into one grant total which was used to determine the type of constitution [19]. A minimal modification on semantic expression of several items of original version was made so as to adapt to local women’s Cantonese habit. Our pilot study demonstrated that the modified version was readable and understandable by Hong Kongers.

### Statistical Analysis

Data were summarized using appropriate descriptive statistics. Univariate analysis on the association between each influencing factor and single TCMC type was performed by Chi-square test, independent Samples *t*-test or One-way ANOVA, as appropriate. Those factors with p-values less than 0.25 in univariate analysis [20] were chosen as candidate variables for stepwise logistic regression analyses to delineate the factors significantly associated with the formation of TCMCs. The severity of complex unbalanced constitutions was described as the total number of the constitution types by individual, and fixed factor ANOVA analyses were used to delineate the factors significantly associated with the complex unbalanced constitution following by univariate analysis. All statistical tests were two-tailed with level of significance set at 0.05.

## Result

### Socio-demographic and physical characteristics

A total of 944 participants who completed the questionnaires were included in the analyses. The frequency distribution of age, area of residence and educational level was no significantly different between the respondents and report for the Hong Kong female population (Table 1). A plurality of participants aged from 45 to 59 years old (64.3%) and had obtained secondary or higher education (73.9%).

**Table 1:** Distribution of subjects of the study in age, geographical distribution and educational level

	**FUR (n = 2154003)**		**Subjects (n = 944)**		**P**
	**N**	**%**	**N**	**%**	
Age (years)					0.227
30-34	324216	15.1	31	3.3	
35-39	328080	15.2	66	7.0	
40-44	330008	15.3	150	15.9	
45-49	356379	16.5	185	19.6	
50-54	324838	15.1	254	26.9	
55-59	259055	12.0	168	17.8	
60-65	231427	10.7	90	9.5	
Home address					0.199
Hong Kong Island	396427	18.4	267	28.3	
Kowloon	625943	29.1	217	23.0	
New Territories	1131633	52.5	460	48.7	
Educational level					0.220
Primary or below	598209	27.8	58	6.1	
Lower secondary	401441	18.6	190	20.1	
US/SF/Diploma/Certificate	745616	34.6	374	39.6	
Sub-degree course or above	382052	17.7	315	33.3	
Unknown	26685	1.2	7	0.7	

**Note**: The data of female resident population in Hong Kong from 2011 Population Census Office, Census and Statistics Department, The Government of the Hong Kong Special Administrative Region [21,22], educational level refers to highest level completed, and P value from Pearson Chi-square compared with Hong Kong female resident population form 30 to 64 years old.

*Abbreviations*: *FUR* female usual residents in Hong Kong, *US* upper secondary, *SF* sixth form.

The studied demographic and reproductive characteristics are shown in Table 2; they had generally healthy standard weight (70.2%), were engaged in a full-time mental work (55.1%), were married (70.7%) and had given births to at least one child (69.9%). As shown in Table 3, Thirty-seven percent of the participants were in good systemic health which were defined as: 1) with no physical diseases (such as hyperthyroidism, rheumatoid arthritis, hyperlipidemia, mild or moderate hypertension, anemia as well as common female diseases such as hyperplasia, uterine fibroids and ovarian cysts); 2) with no mental disease; and 3) did not see doctor due to physical discomfort except for catching cold or body check over the past three months. Sixty-three percent of participants were therefore defined in poor health. With regard to emotional status, about ten percent of participants reported that they had experienced or have been experiencing unpleasant emotional life; fifteen percent reported that their emotional world was in interregnum, the percent of the "ordinary" and "happy" emotion status account for about 72%. Thirty-eight percent reported that they had religious beliefs. Very seldom women reported smoking and drinking habits (2% and 9% respectively). Approximately forty percent reported exercising regularly for at least once per week more than three years.

**Table 2:** Demographic characteristics of the study participants (n = 944)

	**No. (%) or Mean (SD)**
Age (years)	49.59 (7.50) ^ψ^
30 ~ 44	247 (26.2)
45 ~ 59	607 (64.3)
60 ~ 65	90 (9.5)
BMI	22.49 (3.3) ^ψ^
<18.5	66 (7.0)
18.5 ~ 24.9	663 (70.2)
25 ~ 27	91 (9.6)
>27	82 (8.7)
Unknown	42 (4.4)
Occupation	
Full-time working	520 (55.1)
Part-time working	87 (9.2)
House wife/no working	333 (35.3)
No response	4 (0.4)
Working nature	
Manual work	91 (9.6)
M&B	72 (7.6)
Brain work	433 (45.9)
No response	348 (36.9)
Educational level	
Primary school or below	58 (6.1)
Lower secondary	190 (20.1)
US/SS	374 (39.6)
Undergraduate/Sub-degree course	207 (21.9)
Postgraduate or above	108 (11.4)
No response	7 (0.7)
Marital status	
Never married	181 (19.2)
Married/Cohabitating	667 (70.7)
Divorced/Windowed	96 (10.2)
Reproductive history	
Never	284 (30.1)
Yes	660 (69.9)
Parity	
0	284 (30.1)
1	241 (25.5)
2	331 (35.1)
≥3	79 (8.4)
No response	9 (1.0)

**Note**: Data marked with ^ψ^ are presented as mean (standard deviation) and the others are presented as frequency (%).

*Abbreviations*: *M&B* Manual work combined with brain work, *US* upper secondary, *SS* specialized secondary.

**Table 3:** Emotional and healthy status and lifestyle characteristics of the study participants (n = 944)

	**No. (%)**
State of health	
Good	349 (37.0)
Poor	595 (63.0)
Emotional status	
Blank	144 (15.3)
Unhappy (ever)	99 (10.5)
Ordinary	343 (36.3)
happy	348 (36.9)
No response	10 (1.1)
Smoking	
No	925 (98.0)
<5 cigarettes per day	11 (1.2)
6 ~ 10 cigarettes per day	5 (0.5)
11 ~ 20 cigarettes per day	3 (0.3)
Alcohol use	
No	856 (90.7)
<1 time per week	63 (6.7)
1 ~ 2 times per week	18 (1.9)
3 ~ 6 times per week	6 (0.6)
≥1 time per day	1 (0.1)
Religion	
No	580 (61.4)
Yes	364 (38.6)
Exercise regularly	
No	487 (51.6)
Yes	457 (48.4)
Exercise duration (years)	
<3	93 (9.9)
3 ~ 5	116 (12.3)
6 ~ 10	118 (12.5)
>10	74 (7.8)
No response	46 (4.9)

### TCMC types

**Figure 1:**
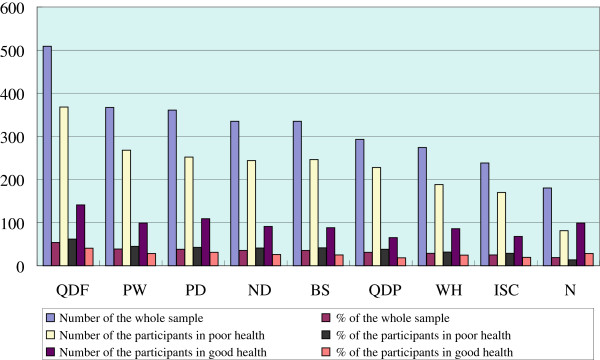
**Distribution of TCMC xtypes among the subjects of the study.** Abbreviations: N, Normality; QDF, Qi-deficiency; QDP, Qi-depressed; PD, Yang-deficiency; ND, Yin-deficiency; PW, Phlegm-wetness; WH, Wetness-heat; BS, Blood stasis; ISC, Inherited special constitution.

**Figure 2:**
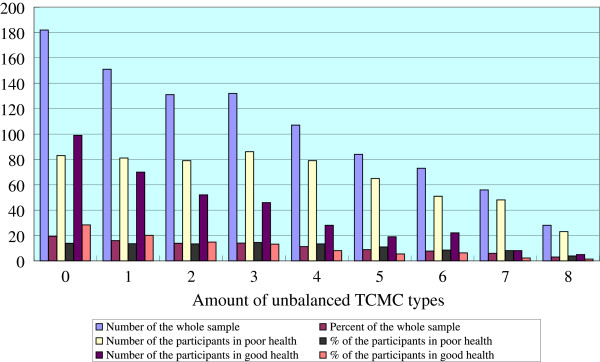
Amount of unbalanced TCMC types among participants.

The number of the participants with each TCMC type and the corresponding percentage distribution were presented in Figure 1. Qi-deficiency constitution was the most frequent TCMC type, and more than half of the whole subjects, followed by phlegm-wetness (38.9%), Yang-deficiency (38.2%), Yin-deficiency (35.5%), Blood-stasis (35.4%), Qi-depressed (31%), Wetness-heat (29%) and Inherited special constitution (25.2%). Normality constitution was identified for up to 19.1%. Although the percentage of each unbalanced TCMC in participants with good health was significantly lower than those with poor health (p < 0.05), the proportion distribution of unbalanced TCMC types was quite similar. Figure 2 shows the distribution of the combined unbalanced TCMC types. Most of them (n = 521, 55.2%), had one to four unbalanced TCMC types at the same time, even with 5 to 8 types (n = 241, 25.5%). The proportion of the participants gradually declined when number of unbalanced TCMC types increased. This trend could be found both in poor health group and in good health group.

### Indicators associated with unbalanced TCMC types

The relationship between the participants’ demographic characteristics, reproductive history, health and emotional status, lifestyle characteristics and the numbers of unbalanced TCMC types were shown in Table 4. The relationship between those independent factors and each single TCMC were individually examined (see Additional file 1). Using those variables with p-values less than 0.25 as candidate variables, eight stepwise logistic models (Table 5) were performed to identify the variables that affect the individual certain TCMC formation. The results showed that (1) Qi-deficiency constitution was significantly associated with healthy status, age, exercise habit and religion; (2) phlegm-wetness constitution was significantly correlated to healthy status, BMI, exercise habit and educational level; (3) Yang-deficiency constitution was closely related with healthy status, BMI and reproductive history; (4) Yin-deficiency constitution was significantly associated with healthy status, age and working nature; (5) Blood-stasis constitution was significantly relevant to healthy status, age and exercise habit; (6) Qi-depressed constitution was closely related to healthy status, age, exercise habit, emotional status; (7) Wetness-heat constitution was significantly associated with age and long-term physical exercise; (8) Normality constitution was significantly correlated to health status, age, educational level and alcohol use.

**Table 4:** Total number of unbalanced TCMC types in different groups with significant influencing factors

**Variables**	**Total number of Unbalanced TCMC types**		**P**
	**Mean**	**SD**	
Age (yrs)			<0.001
30 ~ 44	3.30	2.37	
45 ~ 59	2.81	2.26	
60 ~ 65	2.11	2.34	
Occupation			0.049
Full-time job	3.04	2.32	
Part-time job	2.83	2.17	
HW/UE	2.64	2.34	
Working nature			<0.001
Manual work	2.14	1.98	
M&B	3.18	2.35	
Mental work	3.17	2.32	
Education			<0.001
PS	2.24	2.10	
LS	2.30	2.29	
US/SS	3.05	2.24	
UG/SD	3.17	2.46	
PG	3.12	2.18	
Marital status			0.075
Single	3.18	2.50	
Married/cohabitating	2.83	2.27	
Divorced/windowed	2.56	2.21	
Reproductive history			0.057
Never	3.19	2.42	
Yes	2.74	2.26	
Parity			0.147
1	2.86	2.23	
2	2.76	2.27	
≥3	2.29	2.34	
State of health			0.001
Good	2.14	2.09	
Poor	3.30	2.33	
Emotional status			<0.001
happy	2.48	2.17	
Ordinary	3.12	2.34	
Blank	2.85	2.35	
Unhappy (ever)	3.37	2.52	
Exercise regularly			0.166
No	3.18	2.36	
Yes	2.54	2.26	

Note: Data are listed only when P value from One-way-ANOVA or 2 sample *t*-test is less than 0.25.

*Abbreviations*: *M&B* Manual work combined with brain work, *PS* Primary school or below, *LS* Lower secondary, *US* upper secondary, *SS* specialized secondary, *UG* Undergraduate, *SD* Sub-degree course, *PG* Postgraduate or above.

**Table 5:** Association of TCMC type and significant influencing factors (results of the stepwise logistic regression)

**Variable**	**OR**	**SE**	**95% CI**	**p value**
**Model 1: Outcome: Qi-deficiency constitution**				
**Log-likelihood = 1233.24, chi-square = 69.61(4 d.f.), p < 0.001**				
Age	0.73	0.12	0.57–0.93	0.009
State of health	2.47	0.14	1.87–3.25	<0.001
Exercise regularly	0.61	0.14	0.47–0.80	<0.001
**Model 2: Outcome: Phlegm-wetness constitution**				
**Log-likelihood = 1139.32, chi-square = 62.45(4 d.f.), p < 0.001**				
BMI	1.58	0.10	1.29–1.92	<0.001
Education	1.18	0.07	1.03–1.35	0.015
State of health	2.05	0.15	1.53–2.75	<0.001
Exercise regularly	0.64	0.14	0.49–0.85	0.002
**Model 3: Outcome: Yang-deficiency constitution**				
**Log-likelihood = 1162.98, chi-square = 37.17(3 d.f.), p < 0.001**				
BMI	0.65	0.11	0.52–0.80	<0.001
Reproductive history	0.72	0.15	0.54–0.97	0.028
State of health	1.76	0.15	1.32–2.34	<0.001
**Model 4: Outcome: Yin-deficiency constitution**				
**Log-likelihood = 764.97, chi-square = 34.43(3 d.f.), p < 0.001**				
Age	0.68	0.17	0.49–0.95	0.025
Working nature	1.44	0.13	1.13–1.84	0.003
State of health	2.19	0.18	1.53–3.12	<0.001
**Model 5: Outcome: Blood-stasis constitution**				
**Log-likelihood = 1187.83, chi-square = 38.95(3 d.f.), p < 0.001**				
Age	0.73	0.13	0.57–0.93	0.011
State of health	2.17	0.15	1.61–2.91	<0.001
Exercise regularly	0.75	0.14	0.57–0.97	0.047
**Model 6: Outcome: Qi-depressed constitution**				
**Log-likelihood = 1070.83, chi-square = 84.79(4 d.f.), p < 0.001**				
Age	0.59	0.14	0.45–0.77	<0.001
Emotional status	1.39	0.08	1.20–1.62	<0.001
State of health	2.89	0.17	2.09–4.01	<0.001
Exercise regularly	0.74	0.15	0.55–1.00	0.049
**Model 7: Outcome: Wetness-heat constitution**				
**Log-likelihood = 442.10, chi-square = 12.70(2 d.f.), p < 0.001**				
Age	0.61	0.21	0.41–0.92	0.019
Exercise duration	0.79	0.12	0.63–0.99	0.038
**Model 8: Outcome: Normality Constitution**				
**Log-likelihood = 849.17, chi-square = 56.09(4 d.f.), p < 0.001**				
Age	1.51	0.16	1.11–2.05	0.009
Education	0.80	0.09	0.67–0.94	0.008
State of health	0.37	0.17	0.26–0.52	<0.001
Alcohol use	0.45	0.39	0.21–0.97	0.040

The results from multiple factor ANONA revealed that age, physical exercise, health and emotional status had significant effects on the amount of unbalanced TCMC types (see Table 6). The mean of total numbers of unbalanced TCMC types for subjects who had exercise habit and good systemic health condition were 2.54 and 2.14 respectively, significantly lower than that of those who did not exercise regularly (3.18) and were in poor systemic health (3.30). Those who experienced pleasant emotional life had lower mean amount of unbalanced TCMC types (2.48) than those without. Elderly subjects also had significantly lower mean (2.81 for 45 to 59 years old and 2.11 for 60 to 65 years old) compared with that of 30 to 44 years old (3.30).

**Table 6:** Relationship between TCMC type amount and significant influencing factors (results of multiple factors ANOVA)

**Variables**	**Estimate**	**SE**	**P value**	**Bonferroni’s multiple comparison**
Age			0.002	
30 ~ 44 (1)	1.885	0.698		
45 ~ 59 (2)	1.124	0.675		
60 ~ 65^a^ (3)				(1) > (2) > (3)
State of health			<.001	
Good	-1.21	0.227		
Poor^a^				
Emotional status			0.001	
happy (1)	-1.01	0.406		
Ordinary (2)	-0.05	0.394		
Blank (3)	-0.26	0.609		
Unhappy (ever)^a^ (4)				(1) < (2) = (3) = (4)
Exercise regularly			0.013	
No	0.57	0.228		
Yes^a^				
(Intercept)	1.96	0.94	0.038	

R^2^ = 0.20; Adjusted R^2^ = 0.16; F value = 75.38; df = 18; P < 0.001.

^a^Reference category.

## Discussion

As TCMC could indicate the overall health status of individuals without any serious diseases, and therefore has enormous potential application in treating Not-Yet-Ill (*Zhi Wei Bing* in Chinese, means preventive treatment of diseases) and health care, it is becoming a research and clinical interest. Although a large number of epidemiological investigations on constitution have been performed during past one decade [15,23-29], data on related influencing factors of unbalanced TCMCs are sparse. The present study was one of a limited number of investigations exploring TCMC types among Chinese women in Southern China and associated factors. To our knowledge, this is the first investigation to examine the association between TCMC type and extensive potential influencing factors, rather than only observing the distribution of TCMC types among certain group of people with different demographic characteristics. The findings of this study would help TCM professionals and researchers to strengthen their understanding of the acquired influencing factors of TCMC and thus provide reference for their future studies.

The findings of the present investigation showed that more than eighty percent of adult Chinese women in Hong Kong have unbalanced TCMC, among them more than sixty percent have combined unbalanced constitutions with two or more types. Based on TCMC theory, this finding indicated that they were not in the normal state of health. The most common unbalanced TCMC types among the study subjects were Qi-deficiency, Phlegm-wetness and Yang-deficiency, in both groups with good health and poor health. This finding is basically consistent with that of a previous investigation [27] involved 6525 participants conducted in Guangzhou where climate and environment are similar with Hong Kong. However, the finding of our study differed from previous investigation in Hong Kong which reported nearly half of the respondents in Hong Kong had Normality constitution, and dominant frequency of the unbalanced TCMC types were 9.88% of Yang-deficiency, 9.59% of Qi-deficiency and 8.22% of Wetness-heat constitution respectively, much lower than the distribution percentage in our study [28]. The difference may probably be caused by sampling-related issues. Our study involved the local Chinese women aged from 30 to 65 years old whereas the other study included extended age-range (local women over 15 years old).

The distribution of the dominant unbalanced TCMC types may be closely related to the common life-style of Hong Kong dwellers and Southern-China living environment. Specifically, the formation of Qi-deficiency, based on the theory of "over work damages Qi", might be closely related to prolonged intensive work which is very universal in Hong Kong due to intense competition, fast-paced lifestyle and high pressure from work. Qi-deficiency constitution, characterized by fatigue, low voice, dizziness and prone to cold, is therefore gradually formed [30]. The widespread of Phlegm-wetness constitution among Hong Kong Chinese women might be ascribed to local geographic environment and climate to a large extent. Hong Kong is located at the outlet of the Pear River, by the north of South China Sea and to the south of Nan Ling Mountain, which result in formation of the unique environment and climate. The damp and rainy weather might contribute to the formation of Phlegm-wetness constitution according to TCM philosophy [28]. With regard to the popularity of Yang-deficiency constitution in study subjects, it might be attribute to dietary habit and living habit, which mainly refer to drinking cold drinks and overuse air conditioning whether it is in summer or not. Exposure to these "cold pathogen" for a long period of time may damage individual Yang-qi and thus lead to the formation of Yang-deficiency constitution from the theory of Chinese medicine [28].

The results of the present study showed that the most important associated factor with unbalanced TCMCs is health status. It is well-known that TCM constitution tends to reflect steady state of individual’s health, unbalanced TCMC often represents deteriorated health status even some status diagnosed as "no disease" by western medicine. On the other hand, prolonged chronic illness may conversely affect individual’s TCMC, leading to unbalanced TCMC. In this study, suffering from mild to moderate diseases was associated with some types of unbalanced TCMCs such as Qi-deficiency, Phlegm-wetness, Yang-deficiency, Yin-deficiency, Blood-stasis and Qi-depressed constitutions. This finding confirms the validity of the Chinese Medicine theory of constitution.

In contrast, our study found that physical exercise as the most important protective factor for several certain unbalanced TCMCs such as Qi-deficiency, Phlegm-wetness, Wetness-heat, blood-stasis and Qi-depressed constitutions. The explanation could be as follows: Doing exercise moves Qi in the body and dissolve blood stasis thus improve Blood-stasis and Qi-depressed constitutions. Smooth and fast flow of Qi would help to dispel wetness and thus to prevent the formation of Phlegm-wetness and Wetness-heat constitutions. TCM also holds that "movement could benefit Qi", so regular exercise is effective in improving Qi-deficiency constitution. In addition, the findings indicate that although physical exercise demonstrated protective effect on Wetness-heat constitution formation, only long-term physical exercise could improve wetness-heat condition significantly, the longer exercise duration, the better effect achieved. The reason is that wetness-heat, based on TCM philosophy, is a viscous and lingering pathogen which is very hard to eliminate and therefore usually need long-term treatment and nursing to resolve. However, it does not show any effect on improving Yang-deficiency and Yin-deficiency constitutions which are usually managed via medications or dietary treatment.

"Most overweight people are phlegmatic", as the classical theories of Traditional Chinese Medicine, is proved once again by our study whose result shows overweight is positively associated with phlegm-wetness constitution significantly, and also being consistent with the results of two previous studies [31,32]. Interestingly, although overweight is regarded as an independent risk factor for Phlegm-wetness constitution, it might be a protective factor for Yang-deficiency constitution according to our study result, the high BMI, the less susceptibility for Yang-deficiency constitution. The similar conclusion had been achieved by Prof. Zhu whose study showed that slim body build was significantly positively correlated to Yang-deficiency constitution [33]. One plausible explanation could be that feeling cold is the most distinguished feature to determine Yang-deficiency constitution, while too much subcutaneous fat with the function of keeping body temperature might help fat people improve cold sensation. The findings indicate that high educational level is another precipitating factor for Phlegm-wetness constitution formation, participants received higher education are more likely to emerge Phlegm-wetness constitution than those with low educational level. The possible explanation might be that the women with high educational level are often engaged in senior white-collar jobs with higher income and therefore usually experience sedentary life and intake high quality of diet. According to TCM philosophy, if too many nutrients absorbed into the body exceed the spleen function of transportation and transformation, it would not be transformed into Qi, blood and body fluid, but could be transformed into phlegm and dampness.

Mental work and unpleasant emotional life are also proved to be associated with unbalanced TCMCs. The present study confirms that mental work is an independent risk factor for Yin-deficiency constitution formation. The result demonstrates that those engaged in mental work are prone to get Yin-deficiency constitution than those engaged in manual work, which is entirely consistent with the recognition of the book of *The Yellow Emperor*’*s Inner Classic* which as the earliest classic of TCM believes that over thinking and excessive anxiety may consume Yin and blood secretly and gradually. Based on TCM theory, besides congenital endowment, moodiness is one of most important causes bringing about Qi-depressed condition. Our findings confirm that women have been experiencing less-than-satisfactory emotional life or had experienced less-than-satisfactory emotional life are more prone to Qi-depressed constitution than those with happy emotional life, indicating that negative emotion is indeed a precipitating factor for Qi-depressed constitution.

An important discovery of the present study is that reproductive history is also an independent protective factor for Yang-deficiency constitution, which has never been reported before. Moreover, with the number of full-term pregnancies increasing, the protective effect is more obvious, indicating reproductive history might be favorable for preventing Yang-deficiency formation. But it is unclear whether which is caused by pregnancy, reproduction or other unique postpartum managements. In our opinion, we tend to think that pregnancy and reproduction, as the process consuming Qi and blood, would reflectively switch on body’s self-adjustment function and then stimulate the generation of Yang-qi, leading to the improvement of Yang-deficiency.

To date, whether aging is a precipitating factor or a protective factor for unbalanced TCMC formation is still not sure. Deng claimed that unbalanced TCMCs are more prevalent among old people than the younger [34], however, his investigation only involved 224 participants and without providing any background information of the participants and the details of research methods, so his conclusion is doubtful. The findings of other investigations with large samples were also not totally consistent [15,26-28]. For instance, Chen’s investigation involving 2168 subjects [26] showed that the prevalence of Qi-deficiency, Yang-deficiency, Phlegm-wetness and Blood-stasis among older people is higher than young’s, however, Huang’s results reject accepting Yang-deficiency are more common among elderly than young people [28], the results of Wu’s study involving 2043 subjects [27] and Wang’s study containing 8448 participants [15] demonstrated that there was no significantly difference between old people and young people in the prevalence of Phlegm-wetness. But it needs to be pointed out that all of them only investigated the distribution of unbalanced TCMC types in different age groups, without using logistic regression method to systematically examine the efficacy of age on the formation of TCMC types, so their findings were still not completely convincing. Two studies aiming to explore the associated factors with Phlegm-wetness constitution [31] and Yang-deficiency constitution [33] using logistic regression method also indicated that age was not significantly related to those two types of unbalanced TCMCs. The findings of the present study support aging as a protective factor for the formation of Qi-deficiency, Yin-deficiency, Blood-stasis, Qi-depressed and Wetness-heat constitutions. The potential explanation is that old women usually stay at home and enjoy a leisure life without carrying out heavy work and facing with various pressures, reducing the consumption of Qi and Yin and thus improving Qi and Yin deficiency. With a wealth of experience, elderly are usually good at managing their mood than younger, this would effectively avoid liver-qi stagnation and thus prevent the formation of Qi-depressed and Blood-stasis constitutions. The decline of Wetness-heat constitution incidence among old people may be related to the decrease of their Yang-qi as their age increases, thus contributing to suppress the generation of internal-heat. In summary, so far there is no consensus on the effects of age on unbalanced TCMCs formation, therefore declaring that age is an influencing factor to unbalanced TCMC types might be not justified.

It is universally recognized that smoking is harmful to health, so based on this fact, smoking theoretically should play a role in the formation of unbalanced TCMCs. Wang’s study demonstrated that smoking could promote the formation of Phlegm-wetness constitution [33]. But the present study did not find that smoking would precipitate any types of unbalanced TCMCs formation. A reasonable explanation for this phenomenon is that the sample size with smoking habit is too small and most of them only consume very few cigarettes per day, which might be still not enough to cause the imbalance of their TCMC.

Based on TCM philosophy, the amount of unbalanced TCMC types owned by one person at the same time, representing the complexity of TCMC, usually reflects the severity of his/her constitutional imbalance. Since age, poor health status and exercise habit showed the obvious effect on the formation of most unbalanced TCMC types, and therefore significantly affected the complexity of individuals’ TCMCs. Unpleasant emotional life did not play a significant role in the formation of unbalanced types except for Qi-depressed constitution, but pleasant emotional life did decrease TCMC complexity of the studied participants, further proved the rationality and the science of the theory originated in *The Yellow Emperor*’*s Inner Classic* that emotion is one of the important pathogenic factors.

This study has several limitations. First, the study sample was not recruited via rigorous random sampling method, which may have brought the risk of not being representative enough to generalize the results of all Hong Kong Chinese women. Secondly, although there was no significant difference between the study participants and female long-term residents in age distribution, their age distribution did not match very well, which may be likely affect the objectivity of the results, especially to affect the judgment about the effect of age on the formation of unbalanced TCMCs. Thirdly, other potential influencing factors including dietary habit and work-rest manner which could also affect TCMC formation according to TCM theory, not considered in the study. This may also confound the results.

## Conclusion

Based on the results of this study, some mainly conclusion could be drawn:

First, biased constitutions are common among middle-aged and elderly Chinese women in Hong Kong, indicating it is feasible and necessary to preserve their health and to promote the rehabilitation of their diseases through managing their unbalanced TCM constitutions.

Secondly, poor systemic physical condition is the most important dominating-related factor for the formation of almost all unbalanced TCMC types except for Inherited Special Constitution which is mainly determined by congenital endowments according to TCMC theory. In contrast, regular physical exercise is the most protective factor for most unbalanced TCMC types. This demonstrates that physical condition and exercise history must be treated as the primary influencing factors in such studies about TCMCs.

Thirdly, some special factors may cause certain unbalanced constitutions. For instance, overweight and high educational level contributes to the formation of Phlegm-wetness constitution, mental work contributes to Yin-deficiency constitution and unpleasant emotional life leads to Qi-depressed constitution. This could be explained by TCMC theory, which demonstrates its value.

Last but not least, age as one of the risk factors for unbalanced constitutions in the current TCMC theory might not be justified, indicating some recognition of the current TCMC theory is not entirely correct. Therefore, it is still significant and necessary to confirm the effects of potential factors on unbalanced constitutions formation in further studies with big sample and rigorous design.

## Competing interests

The authors declare that they have no competing interests.

## Authors’ contributions

Youzhi Sun conceived of the design, carried out the study and drafted the manuscript; Jianping Chen was in charge of the study work, advice in the study design; Pei Liu participated in coordination and helped to perform the statistical analysis and manuscript writing; Yi Zhao and Steve An Xue gave expert advice in the study design and participated in manuscript writing; Lei Jia, Yanhua He, Xiao Zheng, Zhiyu Wang and Neng Wang participated in the acquisition of data. All authors read and approved the final manuscript.

## Supplementary Material

Additional file 1 Distribution of TCMC types in different groups with different demographic, emotional, healthy, reproductive and lifestyle influencing factors (N,%). Click here for file
