# Supplementary material for: Characteristics of TCM constitutions of adult Chinese women in Hong Kong and identification of related influencing factors: a cross-sectional survey
Source: J Transl Med. 2014 May 21;12:140. doi: 10.1186/1479-5876-12-140 (PMC4047264; doi:10.1186/1479-5876-12-140)
Supplement: Additional file 1 — Distribution of TCMC types in different groups with different demographic, emotional, healthy, reproductive and lifestyle influencing factors (N,%). [file 1479-5876-12-140-S1.doc]

**Additional file 1: Distribution of TCMC types in different groups with different demographic, emotional, healthy, reproductive and lifestyle influencing factors (N, %**)

|  | QDF(n=509) | | | PW(n=367) | | | PD(n=361) | | | ND(n=335) | | | BS(n=335) | | | QDP(n=293) | | | WH(n=274) | | | N(n=180) | | |
| --- | --- | --- | --- | --- | --- | --- | --- | --- | --- | --- | --- | --- | --- | --- | --- | --- | --- | --- | --- | --- | --- | --- | --- | --- |
|  | No | Yes | P | No | Yes | P | No | Yes | P | No | Yes | P | No | Yes | P | No | Yes | P | No | Yes | P | No | Yes | P |
| Age (yrs) |  |  | 0.016 |  |  | 0.053 |  |  | 0.068 |  |  | 0.014 |  |  | 0.012 |  |  | 0.004 |  |  | <0.001 |  |  | 0.002 |
| 30～44 | 97(22.3) | 150(29.5) |  | 140(24.3) | 107(29.2) |  | 149(25.6) | 98(27.1) |  | 147(24.1) | 100(29.9) |  | 149(24.4) | 98(29.3) |  | 153(23.5) | 94(32.1) |  | 139(20.7) | 108(39.4) |  | 210(27.5) | 37(20.6) |  |
| 45～59 | 288(66.2) | 319(62.7) |  | 373(64.6) | 234(63.8) |  | 375(64.3) | 232(64.3) |  | 393(64.5) | 214(63.9) |  | 391(64.1) | 216(64.7) |  | 426(65.4) | 181(61.8) |  | 457(68.2) | 150(54.7) |  | 493(64.5)) | 114(63.3) |  |
| 60～65 | 50(11.5) | 40(7.8) |  | 64(11.1) | 26(7.0) |  | 59(10.1) | 31(8.6) |  | 69(11.3) | 21(6.2) |  | 70(11.5) | 20(6.) |  | 72(11.1) | 18(6.1) |  | 74(11.0) | 16(5.8) |  | 61(8.0) | 29(16.1) |  |
| BMI |  |  |  |  |  | <0.001 |  |  | 0.001 |  |  |  |  |  |  |  |  |  |  |  |  |  |  |  |
| <18.5 | 32(7.7) | 34(7.0) |  | 48(8.7) | 18(5.1) |  | 32(5.7) | 34(9.9) |  | 45(7.8) | 21(6.4) |  | 38(6.5) | 28(8.8) |  | 47(7.5) | 19(6.8) |  | 47(7.3) | 19(7.3) |  | 59(8.0) | 7(4.3) |  |
| 18.5～24.9 | 305(73.7) | 358(73.4) |  | 423(77.0) | 240(68.0) |  | 398(71.5) | 265(76.8) |  | 428(74.3) | 235(72.1) |  | 434(74.6) | 229(71.6) |  | 459(73.7) | 204(73.1) |  | 475(74.1) | 188(72.0) |  | 540(73.2) | 123(75.0) |  |
| 25～27 | 45(10.9) | 46(9.4) |  | 43(7.8) | 48(13.6) |  | 64(11.5) | 27(7.8) |  | 57(9.9) | 34(10.4) |  | 62(10.7) | 29(9.1) |  | 61(9.8) | 30(10.8) |  | 67(10.5) | 24(9.2) |  | 73(9.9) | 18(11.0) |  |
| >27 | 32(7.7) | 50(10.2) |  | 35(6.4) | 47(13.3) |  | 63(11.3) | 19(5.5) |  | 46(8.0) | 36(11.0) |  | 48(8.2) | 34(10.6) |  | 56(9.0) | 26(9.3) |  | 52(8.1) | 30(11.5) |  | 66(8.9) | 16(9.7) |  |
| Occupation |  |  |  |  |  | 0.040 |  |  |  |  |  | 0.004 |  |  |  |  |  |  |  |  | <0.001 |  |  | 0.058 |
| Full-time job | 232(53.7) | 288(56.7) |  | 300(52.3) | 220(60.1) |  | 321(55.3) | 199(55.3) |  | 312(51.5) | 208(62.3) |  | 337(55.6) | 183(54.8) |  | 359(55.5) | 161(54.9) |  | 339(50.9) | 181(66.1) |  | 435(57.2) | 85(47.5) |  |
| Part-time job | 36(8.3) | 51(10.0) |  | 53(9.2) | 34(9.3) |  | 52(9.0) | 35(9.7) |  | 57(9.4) | 30(9.0) |  | 53(8.7) | 34(10.2) |  | 61(9.4) | 26(8.9) |  | 69(10.4) | 18(6.6) |  | 69(9.1) | 18(10.1) |  |
| HW/UE | 164(38.0) | 169(33.3) |  | 221(38.5) | 112(30.6) |  | 207(35.7) | 126(35.0) |  | 237(39.1) | 96(28.7) |  | 216(35.6) | 117(35.0) |  | 227(35.1) | 106(36.2) |  | 258(38.7) | 75(27.4) |  | 257(33.8) | 76(42.4) |  |
| Working nature |  |  |  |  |  | 0.002 |  |  | 0.036 |  |  | 0.005 |  |  | 0.012 |  |  | 0.234 |  |  | 0.057 |  |  | 0.009 |
| Manual work | 47(17.8) | 44(13.3) |  | 67(19.4) | 24(9.6) |  | 67(18.3) | 24(10.5) |  | 66(18.3) | 25(10.6) |  | 71(18.5) | 20(9.4) |  | 70(16.9) | 21(11.5) |  | 70(17.6) | 21(10.6) |  | 66(13.3) | 25(25.0) |  |
| M&B | 32(12.1) | 40(12.0) |  | 35(10.1) | 37(14.8) |  | 42(11.4) | 30(13.1) |  | 50(13.9) | 22(9.4) |  | 43(11.2) | 29(13.7) |  | 48(11.6) | 24(13.2) |  | 50(12.6) | 22(11.1) |  | 59(11.9) | 13(13.0) |  |
| Brain work | 185(70.1) | 248(74.7) |  | 244(70.5) | 189(75.6) |  | 258(70.3) | 175(76.4) |  | 245(67.9) | 188(80.0) |  | 270(70.3) | 163(76.9) |  | 296(71.5) | 137(75.3) |  | 278(69.8) | 155(78.3) |  | 371(74.8) | 62(62.0) |  |
| Education |  |  | 0.012 |  |  | 0.007 |  |  | 0.152 |  |  | <0.001 |  |  |  |  |  |  |  |  | 0.001 |  |  | <0.001 |
| PS | 33(7.7) | 25(4.9) |  | 43(7.5) | 15(4.1) |  | 37(6.4) | 21(5.8) |  | 42(7.0) | 16(4.8) |  | 42(7.0) | 16(4.8) |  | 42(6.5) | 16(5.5) |  | 46(6.9) | 12(4.4) |  | 42(5.5) | 16(9.0) |  |
| LS | 103(24.0) | 87(17.2) |  | 132(23.1) | 58(15.9) |  | 131(22.7) | 59(16.4) |  | 148(24.5) | 42(12.6) |  | 130(21.5) | 60(18.0) |  | 133(20.6) | 57(19.5) |  | 155(23.3) | 35(12.8) |  | 134(17.6) | 56(31.8) |  |
| US/SS | 151(35.1) | 223(44.0) |  | 217(37.9) | 157(43.0) |  | 224(38.8) | 150(41.7) |  | 234(38.7) | 140(42.0) |  | 235(38.9) | 139(41.7) |  | 258(40.0) | 116(39.7) |  | 258(38.9) | 116(42.5) |  | 320(42.0) | 54(30.7) |  |
| UG/SD | 93(21.6) | 114(22.5) |  | 115(20.1) | 92(25.2) |  | 125(21.7) | 82(22.8) |  | 115(19.0) | 92(27.6) |  | 129(21.4) | 78(23.4) |  | 141(21.9) | 66(22.6) |  | 134(20.2) | 73(26.7) |  | 173(22.7) | 34(19.3) |  |
| PG | 50(11.6) | 58(11.4) |  | 65(11.4) | 43(11.8) |  | 60(10.4) | 48(13.3) |  | 65(10.8) | 43(12.9) |  | 68(11.3) | 40(12.0) |  | 71(11.0) | 37(12.7) |  | 71(10.7) | 37(13.6) |  | 92(12.1) | 16(9.1) |  |
| Marital status |  |  |  |  |  | 0.089 |  |  | 0.032 |  |  | 0.039 |  |  |  |  |  |  |  |  | 0.040 |  |  |  |
| Single | 79(18.2) | 102(20.0) |  | 98(17.0) | 83(22.6) |  | 103(17.7) | 78(21.6) |  | 108(17.7) | 73(21.8) |  | 118(19.3) | 63(18.9) |  | 125(19.2) | 56(19.1) |  | 121(18.1) | 60(21.9) |  | 145(19.0) | 36(20.0) |  |
| Married/Cohabitating | 312(71.7) | 355(69.7) |  | 421(73.0) | 246(67.0) |  | 410(70.3) | 257(71.2) |  | 429(70.4) | 238(71.0) |  | 434(71.1) | 233(69.8) |  | 459(70.5) | 208(71.0) |  | 471(70.3) | 196(71.5) |  | 546(71.5) | 121(67.2) |  |
| Divorced/Windowed | 44(10.1) | 52(10.2) |  | 58(10.0) | 38(10.4) |  | 70(12.0) | 26(7.2) |  | 72(11.8) | 24(7.2) |  | 58(9.5) | 38(11.3) |  | 67(10.3) | 29(9.9) |  | 78(11.6) | 18(6.6) |  | 73(9.5) | 23(12.8) |  |
| Reproductive history |  |  |  |  |  | 0.067 |  |  | 0.007 |  |  | 0.011 |  |  |  |  |  |  |  |  | 0.006 |  |  | 0.196 |
| Never | 124(28.5) | 349(31.4) |  | 161(27.9) | 123(33.5) |  | 157(26.9) | 127(35.2) |  | 166(27.3) | 118(35.2) |  | 184(30.2) | 100(29.9) |  | 196(30.1) | 88(30.0) |  | 184(27.5) | 100(36.5) |  | 237(31.0) | 47(26.1) |  |
| Yes | 311(71.5) | 160(68.6) |  | 416(72.1) | 244(66.5) |  | 426(73.1) | 234(64.8) |  | 443(72.7) | 217(64.8) |  | 426(69.8) | 234(70.1) |  | 455(69.9) | 205(70.0) |  | 486(72.5) | 174(63.5) |  | 527(69.0) | 133(73.9) |  |
| Parity |  |  | 0.097 |  |  |  |  |  | 0.012 |  |  |  |  |  |  |  |  |  |  |  |  |  |  | 0.058 |
| 1 | 107(34.9) | 134(39.0) |  | 147(35.9) | 94(38.8) |  | 150(35.5) | 91(39.7) |  | 160(36.6) | 81(37.9) |  | 156(37.1) | 85(37.0) |  | 164(36.7) | 77(37.7) |  | 174(36.3) | 67(39.0) |  | 195(37.6) | 46(34.8) |  |
| 2 | 154(50.2) | 177(51.5) |  | 214(52.3) | 117(48.3) |  | 209(49.5) | 122(53.3) |  | 221(50.6) | 110(51.4) |  | 210(49.9) | 121(52.6) |  | 232(51.9) | 99(48.5) |  | 241(50.3) | 90(52.3) |  | 269(51.8) | 62(47.0) |  |
| ≥3 | 46(14.9) | 33(9.5) |  | 48(11.7) | 31(12.8) |  | 63(15.0) | 16(7.0) |  | 56(12.8) | 23(10.7) |  | 55(13.1) | 24(10.4) |  | 51(11.4) | 28(13.7) |  | 64(13.4) | 15(8.7) |  | 55(10.6) | 24(18.2) |  |
| State of health |  |  | <0.001 |  |  | <0.001 |  |  | 0.001 |  |  | <0.001 |  |  | <0.001 |  |  | <0.001 |  |  | 0.023 |  |  | <0.001 |
| Good | 208(47.8) | 141(27.7) |  | 250(43.3) | 99(27.0) |  | 240(41.2) | 109(30.2) |  | 258(42.4) | 91(27.2) |  | 261(42.8) | 88(26.3) |  | 284(43.6) | 65(22.2) |  | 263(39.3) | 86(31.4) |  | 250(32.7) | 99(55.0) |  |
| Poor | 227(52.2) | 368(72.3) |  | 327(56.7) | 268(73.0) |  | 343(58.8) | 252(69.8) |  | 351(57.6) | 244(72.8) |  | 349(57.2) | 246(73.7) |  | 367(56.4) | 228(77.8) |  | 407(60.7) | 188(68.6) |  | 514(67.3) | 81(45.0) |  |
| Emotional status |  |  | 0.006 |  |  | 0.013 |  |  |  |  |  | 0.124 |  |  | 0.021 |  |  | <0.001 |  |  |  |  |  | 0.099 |
| happy | 185(42.9) | 163(32.4) |  | 236(41.3) | 112(30.9) |  | 227(39.2) | 121(34.1) |  | 234(38.9) | 114(34.2) |  | 242(40.0) | 106(32.2) |  | 265(41.1) | 83(28.7) |  | 253(38.2) | 95(34.9) |  | 269(35.6) | 79(44.1) |  |
| Ordinary | 138(32.0) | 205(40.8) |  | 198(34.7) | 145(39.9) |  | 199(34.4) | 144(40.6) |  | 204(33.9) | 139(41.7) |  | 215(35.5) | 128(38.9) |  | 229(35.5) | 114(39.4) |  | 238(36.0) | 105(38.6) |  | 288(38.1) | 55(30.7) |  |
| Blank | 67(15.5) | 77(15.3) |  | 83(14.5) | 61(16.8) |  | 92(15.9) | 52(14.6) |  | 98(16.3) | 46(13.8) |  | 95(15.7) | 49(14.9) |  | 104(16.1) | 40(13.8) |  | 101(15.3) | 43(15.8) |  | 114(15.1) | 30(16.8) |  |
| Unhappy (ever) | 41(9.5) | 58(11.5) |  | 54(9.5) | 45(12.4) |  | 61(10.5) | 38(10.7) |  | 65(10.8) | 34(10.2) |  | 53(8.8) | 46(14.0) |  | 47(7.3) | 52(18.0) |  | 70(10.6) | 29(10.7) |  | 84(11.1) | 15(8.4) |  |
| Smoking |  |  |  |  |  |  |  |  |  |  |  |  |  |  |  |  |  |  |  |  |  |  |  |  |
| No | 428(98.4) | 497(97.6) |  | 565(97.9) | 360(98.1) |  | 573(98.3) | 352(97.5) |  | 596(97.9) | 329(98.2) |  | 597(97.9) | 328(98.2) |  | 636(97.7) | 289(98.6) |  | 655(97.8) | 270(98.5) |  | 748(97.9) | 177(98.3) |  |
| Yes | 7(1.6) | 12(2.4) |  | 12(2.1) | 7 (1.9) |  | 10(1.7) | 9(2.5) |  | 13(2.1) | 6(1.8) |  | 13(2.1) | 6(1.8) |  | 15(2.3) | 4(1.4) |  | 15(2.2) | 4(1.5) |  | 16(2.1) | 3(1.7) |  |
| Alcohol use |  |  | 0.090 |  |  |  |  |  |  |  |  | 0.177 |  |  |  |  |  |  |  |  |  |  |  | 0.053 |
| No | 402(92.4) | 454(89.2) |  | 527(91.3) | 329(89.6) |  | 524(89.9) | 332(92.0) |  | 558(91.6) | 298(89.0) |  | 556(91.1) | 300(89.8) |  | 592(90.9) | 264(90.1) |  | 610(91.0) | 246(89.8) |  | 686(89.8) | 170(94.4) |  |
| Yes | 33(7.6) | 55(10.8) |  | 50(8.7) | 38(10.4) |  | 59(10.1) | 29(8.0) |  | 51(8.4) | 37(11.0) |  | 54(8.9) | 34(10.2) |  | 59(9.1) | 29(9.9) |  | 60(9.0) | 28(10.2) |  | 78(10.2) | 10(5.6) |  |
| Religion |  |  | 0.012 |  |  | 0.011 |  |  | 0.058 |  |  |  |  |  |  |  |  | 0.060 |  |  | 0.168 |  |  | 0.035 |
| No | 286(65.7) | 294(57.8) |  | 373(64.6) | 207(56.4) |  | 372(63.8) | 208(57.6) |  | 380(62.4) | 200(59.7) |  | 375(61.5) | 205(61.4) |  | 413(63.4) | 167(57.0) |  | 421(62.8) | 159(58.0) |  | 457(59.8) | 123(68.3) |  |
| Yes | 149(34.3) | 215(42.2) |  | 204(35.4) | 160(43.6) |  | 211(36.2) | 153(42.4) |  | 229(37.6) | 135(40.3) |  | 235(38.5) | 129(38.6) |  | 238(36.6) | 126(43.0) |  | 249(37.2) | 115(42.0) |  | 307(40.2) | 57(31.7) |  |
| Exercise regularly |  |  | <0.001 |  |  | 0.001 |  |  | 0.115 |  |  | 0.054 |  |  | 0.011 |  |  | 0.005 |  |  | 0.001 |  |  | 0.193 |
| No | 193(44.4) | 294(55.8) |  | 273(47.3) | 214(58.3) |  | 289(49.6) | 198(54.8) |  | 300(49.3) | 187(55.8) |  | 296(48.5) | 191(57.2) |  | 316(48.5) | 171(58.4) |  | 323(48.2) | 164(59.9) |  | 402(52.6) | 85(47.2) |  |
| Yes | 242(55.6) | 215(42.2) |  | 304(52.7) | 153(41.7) |  | 294(50.4) | 163(45.2) |  | 309(50.7) | 148(44.2) |  | 314(51.5) | 143(42.8) |  | 335(51.5) | 122(41.6) |  | 347(51.8) | 110(40.1) |  | 362(47.4) | 95(52.8) |  |
| Exercise duration (yrs) |  |  |  |  |  |  |  |  |  |  |  |  |  |  |  |  |  |  |  |  | 0.019 |  |  |  |
| <3 | 46(22.3) | 47(24.1) |  | 63(23.8) | 30(22.1) |  | 59(22.7) | 34(24.1) |  | 65(24.4) | 28(20.7) |  | 67(24.7) | 26(20.0) |  | 69(23.8) | 24(21.6) |  | 61(20.4) | 32(31.4) |  | 78(24.1) | 15(19.2) |  |
| 3～5 | 65(31.6) | 51(26.2) |  | 75(28.3) | 41(30.1) |  | 81(31.2) | 35(24.8) |  | 79(29.7) | 37(27.4) |  | 77(28.4) | 39(30.0) |  | 85(29.3) | 31(27.9) |  | 82(27.4) | 34(33.3) |  | 88(27.2) | 28(35.9) |  |
| 6～10 | 55(26.7) | 63(32.3) |  | 78(29.4) | 40(29.4) |  | 75(28.8) | 43(30.5) |  | 71(26.7) | 47(34.8) |  | 78(28.8) | 40(30.8) |  | 82(28.3) | 36(32.4) |  | 98(32.8) | 20(19.6) |  | 98(30.3) | 20(25.6) |  |
| >10 | 40(19.4) | 34(17.4) |  | 49(18.5) | 25(18.4) |  | 45(17.3) | 29(20.6) |  | 51(19.2) | 23(17.0) |  | 49(18.1) | 25(19.2) |  | 54(18.6) | 20(18.0) |  | 58(19.4) | 16(15.7) |  | 59(18.3) | 15(19.2) |  |

**Note**: P value from Pearson Chi-square are listed when P<0.25.

**Abbreviations**: N, Normality; QDF, Qi-deficiency; QDP, Qi-depressed; PD, Yang-deficiency; ND, Yin-deficiency; PW, Phlegm-wetness; WH, Wetness-heat; BS, Blood stasis; ISC, Inherited special constitution. HW, House wife; UE, Unemployment; M&B, Manual work combined with brain work; PS, Primary school or below; LS, Lower secondary; US, upper secondary; SS, specialized secondary; UG, Undergraduate; SD, Sub-degree course; PG, Postgraduate or above.
